# Rare haplotype load as marker for lethal mutagenesis

**DOI:** 10.1371/journal.pone.0204877

**Published:** 2018-10-03

**Authors:** Josep Gregori, María Eugenia Soria, Isabel Gallego, Mercedes Guerrero-Murillo, Juan Ignacio Esteban, Josep Quer, Celia Perales, Esteban Domingo

**Affiliations:** 1 Liver Unit, Liver Disease Laboratory-Viral Hepatitis, Internal Medicine Department, Vall d’Hebron Institut Recerca (VHIR)-Hospital Universitari Vall d’Hebron (HUVH), Barcelona, Spain; 2 Centro de Investigación Biomédica en Red de Enfermedades Hepáticas y Digestivas (CIBERehd) del Instituto de Salud Carlos III, Madrid, Spain; 3 Roche Diagnostics, S.L., Sant Cugat del Vallés, Barcelona, Spain; 4 Centro de Biología Molecular “Severo Ochoa” (CSIC-UAM), Consejo Superior de Investigaciones Científicas (CSIC), Campus de Cantoblanco, Madrid, Spain; 5 Universitat Autónoma de Barcelona, Barcelona, Spain; Institut Pasteur, FRANCE

## Abstract

RNA viruses replicate with a template-copying fidelity, which lies close to an extinction threshold. Increases of mutation rate by nucleotide analogues can drive viruses towards extinction. This transition is the basis of an antiviral strategy termed lethal mutagenesis. We have introduced a new diversity index, the rare haplotype load (RHL), to describe NS5B (polymerase) mutant spectra of hepatitis C virus (HCV) populations passaged in absence or presence of the mutagenic agents favipiravir or ribavirin. The increase in RHL is more prominent in mutant spectra whose expansions were due to nucleotide analogues than to multiple passages in absence of mutagens. Statistical tests for paired mutagenized versus non-mutagenized samples with 14 diversity indices show that RHL provides consistently the highest standardized effect of mutagenic treatment difference for ribavirin and favipiravir. The results indicate that the enrichment of viral quasispecies in very low frequency minority genomes can serve as a robust marker for lethal mutagenesis. The diagnostic value of RHL from deep sequencing data is relevant to experimental studies on enhanced mutagenesis of viruses, and to pharmacological evaluations of inhibitors suspected to have a mutagenic activity.

## Introduction

The mutant spectra of RNA viruses are a reflection of their evolutionary history, as well as important determinants of virus adaptability. Concerning control of viral diseases, mutant spectrum dynamics is an obstacle for the efficacy of therapeutic interventions due to selection of treatment-escape viral mutants. The antiviral agents to combat RNA viruses include those directed to specific viral targets [direct-acting antiviral agents (DAAs)], and those that inhibit cellular functions needed for the completion of the virus life cycle. The viral RNA-dependent RNA polymerase (RdRp) is the target of several effective antiviral agents. Some of them, notably base or nucleoside analogues, are intracellularly converted into their active nucleotide counterparts. The discovery that ribavirin (1-*β*-D-ribofuranosyl-1-*H*-1,2,4-triazole-3-carboxamide) is mutagenic for poliovirus [1] introduced a new perspective in the antiviral mechanism of some nucleotide analogues. Three alternative ─not mutually exclusive─ mechanisms of anti-RdRp activity by nucleotide analogues have been described: RNA chain termination, inhibition of RNA synthesis without chain termination, and inhibition associated with viral genome mutagenesis.

Nucleotide analogue-induced mutagenesis is equivalent to a decrease of copying fidelity by the viral RdRp. Quasispecies theory predicts a maximum amount of genetic information that can be transmitted for a given average copying fidelity. This concept is mathematically formulated in the form of an error threshold relationship. An increase in mutation rate drives the population across the error threshold, into error catastrophe, equated with loss of inheritable information [2, 3]. The error threshold applies to finite populations in variable fitness landscapes, and its position in a fidelity scale depends also on the degree of adaptation of the mutant ensemble to the environment [3]. The error threshold concept has found experimental support in studies on the negative effects of chemical mutagenesis on the survival of RNA viruses ([4–8], among other studies). The convergence of theoretical and experimental results opened the way to lethal mutagenesis as an antiviral strategy [9].

The licensed antiviral nucleoside analogues favipiravir (T-705; 6-fluoro-3-hydroxy-2-pirazinecarboxamide) and ribavirin are mutagenic for several RNA viruses. Ribavirin has been used as antiviral agent for decades [10, 11], and only recently shown to be mutagenic for several RNA viruses [1, 12, 13]. Favipiravir has been licensed as an anti- influenza agent in Japan having potent antiviral activity against different influenza virus strains (types A, B and C) including those resistant to neuraminidase and M2 inhibitors [14]. Favipiravir has also been effective to inhibits the replication of other RNA viruses in vitro and in animal models, including flavi-, noro- alpha-, bunya-, arena-, filovirus and other RNA virus for which no antiviral therapy is currently available [reviewed in (15, 16)]. Favipiravir in converted intracellularly into the ribofuranosyl 5’-triphosphate metabolite (favipiravir-RTP) and in this form it can be recognized as a pseudopurine by the RdRp [14, 17]. Its selective inhibition of RdRp implicates a wider anti-viral spectrum with a limited cell damage compared with other mutagens such as Ribavirin.

Incorporation of favipiravir-RTP in the nascent viral RNA could result in lethal mutagenesis, as has been proposed for influenza virus [18], norovirus [7], hepatitis C virus (HCV) [19]; foot-and-mouth disease virus [20], West Nile virus [21], Dengue virus [22] and Ebola virus [8]; coxsackievirus B3 [23]. It is not clear whether favipiravir acts as RNA chain terminator, inhibitor, mutagen or by a combination of these mechanisms; its dominant mode of action may depend on the virus-host system and concentration of the active form. In order to cause lethal mutagenesis, favipiravir-RTP needs to be incorporated into the RNA without causing immediate chain termination. It is possible that both lethal mutagenesis and chain termination occur depending on the available concentration of favipiravir-RTP. It has been hypothesized that incorporation of low levels of favipiravir-RTP could result in full-length extension of the viral RNA, leading to lethal mutagenesis and lower infectivity [24].

The standard way to distinguish an RNA virus mutagen from a non-mutagenic inhibitor, is that a mutagenic inhibitor promotes an increase of mutant spectrum diversity, and a decrease of the virus specific infectivity (defined as the ratio of the number of infectious units to the amount of viral RNA in the virus population) (reviewed in [25, 26]). The application of deep sequencing to the analysis of viral populations has introduced several new diversity indices that allow a more detailed description of mutant spectra [27–29]. Some diversity indices were adopted from ecology, and are classified in three groups: incidence (based on the count of entities in a multiple alignment of haplotypes), abundance (that considers both, counts of entities and their frequency), and functional (based on differences among the observed haplotypes) [27]. The value of alternative diversity indices to diagnose the mechanism underlying the expansion of mutant spectra is an unsolved issue.

We have adapted a HCV serial passage design to study the genetic and phenotypic diversification of HCV in Huh-7.5 reporter cells in absence of cellular evolution [30–32]. The parental (plasmid-derived) HCV population was passaged in absence or presence of ribavirin or favipiravir. Populations whose mutant spectrum was expanded in absence of drugs were also subjected to mutagenesis. The design produced several HCV populations for comparative mutant spectrum analyses. NS5B amplicons were analyzed to quantify mutant spectrum complexity. We describe a new diversity index, the rare haplotype load (RHL), and show that its variation outstands among that of other diversity indices to characterize mutant spectra in their transition into error catastrophe. RHL may help in the understanding of quasispecies dynamics, and in the clarification of the mechanisms of action of antiviral agents.

## Results

### The rare haplotype load of hepatitis C virus populations

**Fig 1:**
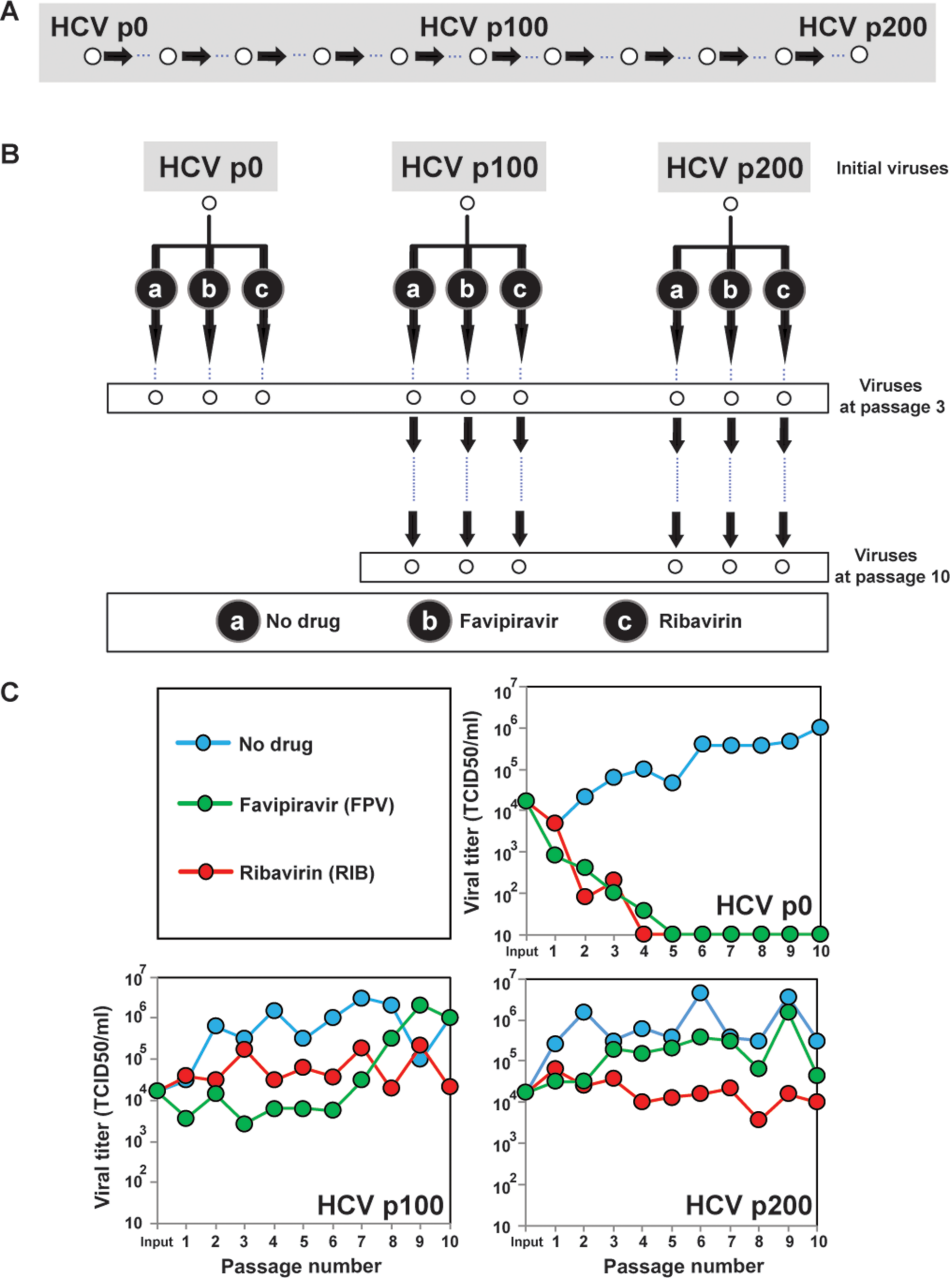
Experimental design and infectious HCV progeny production in absence or presence of favipiravir or ribavirin. (A) Passage of HCV p0 in Huh-7.5 reporter cells to derive high fitness HCV p100 and HCV p200 populations. (B) Serial passages of HCV p0, HCV p100 and HCV p200 in absence of drugs (No drug) or the presence of 400 μM favipiravir, or 100 μM ribavirin. (C) Infectious progeny production during 10 serial passages in absence or presence of the drugs. Details on the infections are given in Materials and Methods.

HCV RNA expressed from plasmid Jc1FLAG2(p7-nsGluc2A) (genotype 2a) [33] was transfected into Huh-7 Lunet cells and amplified in Huh-7.5 cells to produce the initial virus population HCV p0 [30]. HCV p0 was subjected to 200 serial passages in Huh-7.5 reporter cells in the absence of any drug. The populations at passage 100 (HCV p100) and at passage 200 (HCV p200) displayed increased replication in Huh-7.5 reported cells [31, 32]. HCV p0, HCV p100 and HCV p200 were further passaged either in the absence of any drug or in the presence of favipiravir or ribavirin (Fig 1A). Infectious progeny levels were those expected from previous quantifications of inhibition of HCV p0 by favipiravir [19] and ribavirin [34]; the sustained HCV p100 and HCV p200 production in the presence of the drugs is expected from the fitness-associated HCV resistance to antiviral agents [31, 32, 35] (Fig 1B).

Intracellular viral RNA was sequenced in MiSeq with 2x300 mode with v3 chemistry, and fastq files were analyzed as previously described [27, 29] to obtain forward and reverse consensus haplotypes with abundances not below 0.1%, median coverage 147,000 reads, interquartile range (IQR) 75570–226100. The fasta files obtained for each sample were further subjected to DSFT for diversity indices computation. The resulting median coverage was of 139200 with IQR 71,480–210,600 reads.

We introduce the rare haplotype load (RHL) as a new diversity index which may be considered intermediate between incidence and abundance indices. In the context of this work we define as rare those haplotypes with abundances below a given threshold (1%), and as load the fraction of molecules in the quasispecies belonging to these haplotypes. Translating this concept to next generation sequencing is not an easy task as we wish to take into account full reads in the range of abundances below the instrument noise level. Our approach consisted in taking all reads corresponding to haplotypes common to the forward and reverse strand with no previous abundance filtering and computing the RHL as the fraction of reads belonging to haplotypes with abundances below 1%. We suppose that technical noise affects equally all samples in the experiment and that the distinctive effect would be caused by the treatment. This index was not submitted to any sample size correction.

### Comparison of RHL with other diversity indices

**Fig 2:**
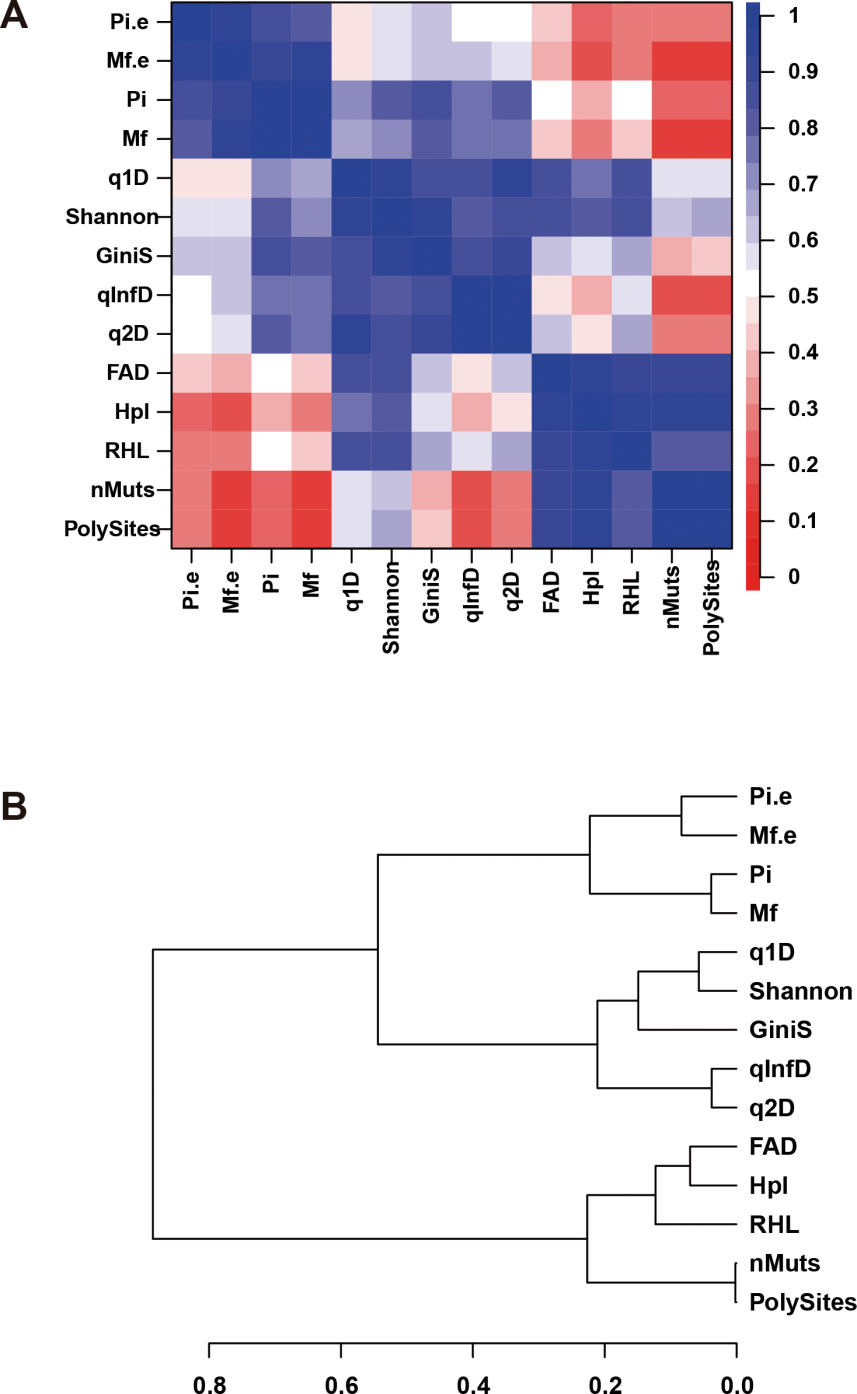
Correlation among diversity. (A) Plot illustrating the correlation between diversity indices in this study. The correspondences between colors and correlation values is shown on the right bar. (B) Hierarchical clustering of diversity indices computed with the square root of one minus the correlation matrix, as measure of dissimilarity.

A full set of diversity indices was computed for each sample: Hpl, number of haplotypes; PolySites, number of polymorphic sites; nMuts, number of mutations; Shannon, Shannon entropy; GiniS, Gini-Simpson index; q1D, Hill number of order 1; q2D, Hill number of order 2; qInfD, Hill number of order infinity; FAD, functional attribute diversity; Mf.e, mutation frequency by entity; Pi.e, nucleotide diversity by entity; Pi, nucleotide diversity, and Mf, mutation frequency. The correlation among all indices (including RHL) resulting from all samples in the experimental design, shows a structure with three groups (Fig **2**) G1: RHL, Hpl, FAD, nMuts and PolySites; G2: Shannon, GiniS, q1D, q2D and qInfD; and G3: Mf.e, Pi.e, Mf and Pi. The three indices more correlated to RHL are Hpl 0.895, FAD 0.854 and Shannon 0.826. RHL falls within the group of incidence indices, but it results from the aggregation of abundances. Its high correlation with Shannon and q1D denotes properties of abundance-based indices, while its high correlation with FAD confers to RHL properties of functional incidence. These three properties were expected from the definition of RHL, and the correlations provide an empirical prove of the computations adequacy.

### Association tests

Table 1 summarizes the results of the association tests of each diversity index considered, including RHL, to mutagenicity, for each drug (favipiravir and ribavirin) sorted by decreasing order of standardized effect. No distinction has been made of amplicon or treatment length. Hence the results represent averaging over the full NS5B region sequenced, and over the two treatment lengths. The RHL is the index with the highest standardized effect among all in both mutagenic treatments, with adjusted p-values of the order of 10^−4^. Top indices are also FAD, Hpl, Shannon, nMuts, and PolySites. No significant association is found in neither treatment, for Mf and Pi, both at the abundance and at the entity level. RHL is still the top indicator when distinguishing among amplicons or treatment length, followed by FAD and Hpl.

**Table 1:** Wilcoxon signed rank test of paired samples, treatment vs control. Rows sorted in descending order of standardized effect. Estimate: median of treatment difference, SD: standard deviation, StdEffect: standardized effect (median divided by sd), p.value: Wilcoxon test p-value, adj.pv: multiple test adjusted p-value by the Bonferroni method.

	Ribavirin						Favipiravir				
	Estimate	SD	StdEffect	p.value	adj.pv		Estimate	SD	StdEffect	p.value	adj.pv
**RHL**	1,49E+01	5,09E+00	2,920	3,05E-05	4,27E-04	**RHL**	1,81E+01	3,86E+00	4,680	3,05E-05	4,27E-04
**nMuts**	2,05E+01	1,00E+01	2,040	3,58E-04	5,01E-03	**Shannon**	8,76E-01	4,22E-01	2,080	3,05E-05	4,27E-04
**Hpl**	2,25E+01	1,11E+01	2,020	3,61E-04	5,06E-03	**FAD**	1,74E+01	9,33E+00	1,860	6,10E-05	8,54E-04
**PolySites**	2,00E+01	9,92E+00	2,010	3,58E-04	5,01E-03	**Hpl**	2,75E+01	1,54E+01	1,790	5,45E-04	7,62E-03
**FAD**	1,21E+01	6,55E+00	1,850	3,05E-05	4,27E-04	**GiniS**	2,15E-01	1,39E-01	1,550	2,14E-04	2,99E-03
**Shannon**	6,99E-01	3,89E-01	1,800	3,05E-05	4,27E-04	**q1D**	5,18E+00	3,33E+00	1,550	3,05E-05	4,27E-04
**GiniS**	1,62E-01	1,27E-01	1,280	3,05E-04	4,27E-03	**nMuts**	2,60E+01	1,77E+01	1,470	1,59E-03	2,22E-02
**q1D**	4,04E+00	3,95E+00	1,020	3,05E-05	4,27E-04	**PolySites**	2,40E+01	1,71E+01	1,400	1,18E-03	1,65E-02
**qInfD**	7,74E-01	7,64E-01	1,010	5,80E-04	8,12E-03	**q2D**	1,56E+00	1,74E+00	0,901	3,05E-04	4,27E-03
**q2D**	1,58E+00	2,06E+00	0,766	3,05E-04	4,27E-03	**qInfD**	6,71E-01	9,05E-01	0,741	1,31E-03	1,84E-02
**Mf.e**	6,52E-04	1,55E-03	0,420	5,35E-02	7,49E-01	**Pi**	1,22E-03	2,04E-03	0,596	1,77E-02	2,47E-01
**Pi**	5,44E-04	1,34E-03	0,407	4,73E-02	6,62E-01	**Pi.e**	5,73E-04	1,40E-03	0,410	8,44E-02	1,00E+00
**Mf**	5,38E-04	1,37E-03	0,393	2,77E-02	3,88E-01	**Mf**	7,04E-04	2,32E-03	0,304	3,19E-02	4,46E-01
**Pi.e**	3,32E-04	1,17E-03	0,284	1,51E-01	1,00E+00	**Mf.e**	3,85E-04	1,72E-03	0,223	2,11E-01	1,00E+00

### Logistic regression

**Fig 3:**
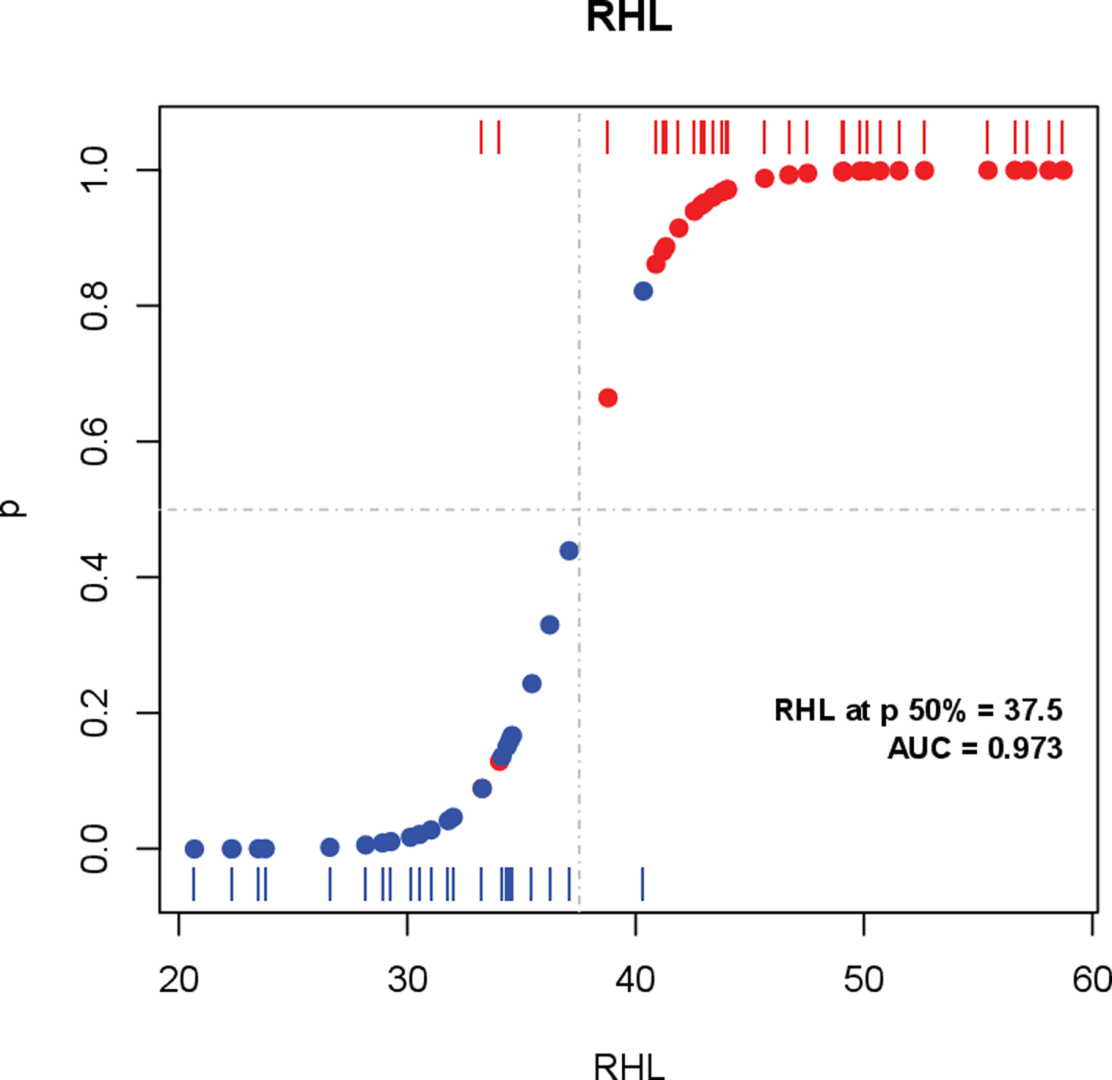
Logistic regression plot of mutagenicity over RHL. Red bars at the top depict the values of RHL for HCV samples subject to mutagenesis. Blue bars at the bottom depict RHL values for control samples. Corresponding blue and red dots on the fitted logistic curve show the predicted probability of mutagenesis for each sample, with a predicted 50% probability at a RHL value of 37.5%. Area under the ROC curve 0.973. Only two samples under mutagenic drug treatment and one control sample were mistakenly classified.

As a further step towards characterizing RHL as mutagenicity marker, samples were relabeled as under mutagenicity (Mut) if treated with favipiravir or ribavirin, or not mutagenized (control) for passages in absence of drug. Each group includes the variability due to the factors amplicon, drug, and treatment length. Then a univariate logistic regression was fit with each of the diversity indices, including RHL (Figs 3 and S1). The regression to RHL resulted in the best value of the Aikake Information content (AIC), the highest area under the ROC curve (AUC), and the lowest leave-one out cross-validation (LOOCV) error rate to classification (Table 2). According to the LOOCV error rate the predictive capacity of these indices follows the order RHL, Hpl, PolySites, nMuts and FAD (S2 Fig).

**Table 2:** Results of the logistic regressions. Dev: residual deviance. AIC: Aikake Information Content. Sensit: Sensitivity. Specif: Specificity. AUC: area under the ROC curve. Err: classification error rate. LOOCV: Leave-one-out cross-validation error rate.

Index	Dev	AIC	Sensit	Specif	AUC	Err	TenFoldCV	LOOCV
**RHL**	19,6	23,6	0,933	0,958	0,973	0,056	0,057	0,055
**Hpl**	24,2	28,2	0,867	0,917	0,968	0,111	0,123	0,111
**PolySites**	44,2	48,2	0,800	0,875	0,899	0,167	0,176	0,165
**nMuts**	45,3	49,3	0,800	0,875	0,897	0,167	0,176	0,172
**FAD**	36,5	40,5	0,867	0,792	0,928	0,167	0,182	0,181
**Shannon**	49,7	53,7	0,833	0,750	0,863	0,204	0,208	0,204
**q1D**	50,4	54,4	0,800	0,792	0,863	0,204	0,215	0,221
**q2D**	62,5	66,5	0,633	0,833	0,738	0,278	0,290	0,278
**qInfD**	62,8	66,8	0,633	0,833	0,724	0,278	0,298	0,312
**GiniS**	62,4	66,4	0,767	0,542	0,738	0,333	0,345	0,367
**Pi.e**	74,1	78,1	1,000	0,000	0,557	0,444	0,485	0,465
**Pi**	72,3	76,3	0,700	0,292	0,597	0,482	0,539	0,516
**Mf**	71,4	75,4	0,633	0,417	0,589	0,463	0,520	0,529
**Mf.e**	73,4	77,4	0,933	0,208	0,562	0,389	0,494	0,571

When this analysis was performed separately for each treatment length, for 10 passes both RHL, Shannon and q1D resulted in the lowest values of AIC and LOOCV, for 3 passes RHL, FAD and Hpl resulted in the lowest values of AIC and LOOCV (S3 and S4 Figs). No multivariate logistic regression model resulted better that RHL as a single predictor in the whole dataset.

These results prove that within the experimental design of this study RHL is the most sensitive diversity index for predicting mutagenic effects, with independence of factors such as amplicon, base line passage, drug, and treatment length.

### In-silico study to test the robustness and unbiasedness of RHL

To study the robustness of RHL and its possible dependence on sample size, an in-silico study on the full set of fasta files with no exclusion was performed. Robustness was evaluated by comparing RHL with the number of haplotypes, Hpl. According to the main statistics of the distribution of median values obtained after 2000 simulation cycles on each fasta file, RHL is on average five times less variable than Hpl, in terms of the coefficient of variation (CV) and of interquartile dispersion (QD) (**Table 3**). The main statistics of the relative error of the median value of RHL in the sample replicates indicate a median error of 0.04% with a maximum of 1.99%. This confirms that the observed value of RHL in a sample is unbiased and not influenced by the sample size. No sample size correction was needed for RHL, contrary to most diversity indices [27, 29]. Thus, RHL is far more stable and robust than Hpl and other diversity indices to characterize a virus transition into error catastrophe with deep sequencing data.

**Table 3:** Results of the in-silico study to characterize the biasedness and robustness of RHL with respect to Hpla. The table provides a summary of distributional values for the % error with respect to the true value of RHL (top row); the coefficient of variation (CV) observed for RHL and Hpl, rows second and third; the ratio of both CVs in row fourth; the interquartile deviation (QD) observed for RHL and Hpl, rows fifth and sixth; and the ratio of both QDs in the last row.

	Min.	Q1	Median	Mean	Q3	Max.
**RHL.err %**	0,00	0,02	0,04	0,25	0,32	1,99
**RHL.cv**	0,0048	0,0077	0,0098	0,0109	0,0134	0,0211
**Hpl.cv**	0,0279	0,0399	0,0443	0,0505	0,0548	0,1086
**CV.ratio**	2,1	3,2	4,7	5,3	6,9	13,1
**RHL.qd**	0,0057	0,0097	0,0120	0,0147	0,0184	0,0378
**Hpl.qd**	0,0000	0,0488	0,0606	0,0637	0,0741	0,1538
**QD.ratio**	0,0	2,9	5,1	5,2	7,0	13,7

## Discussion

Comparison of diversity indices for complexity evaluation of mutant spectra of HCV populations has unveiled that RHL is a reliable index to diagnose mutant spectrum expansions associated with a mutagenic treatment. Previous studies with HCV quantified average 5.1-fold (range 3.8–6.6) increases of mutation frequency following 200 serial large population passages in Huh-7.5 cells, and 3.5-fold (range 1.6–5.6) increases as a result of up to 5 serial passages in the presence of favipiravir or ribavirin [19, 32, 34]. Despite comparable or even larger mutation frequency increases associated with multiple passages compared with mutagenic treatments, RHL stood as a reliable, robust and unbiased marker for lethal mutagenesis. RHL is less influenced by standard serial passages.

A salient informative role of RHL can be interpreted in the light of current evidence of the molecular mechanisms that underlie the transition of RNA viruses towards an extinction threshold. One event is suppression of viable genome replication by defective genomes that are produced as a result of mutagenesis [36–38]. This is an extension to mutagenized populations of the capacity of mutant spectra to suppress replication of high fitness cognate populations [39]. Mutant-dependent interference was formulated as the lethal defection model of virus extinction [40] that has as one of its consequences that during mutagenesis infectivity is lost earlier that the capacity of viral RNA to replicate, thus leading to decrease of specific infectivity [19, 20, 34, 41, 42]. Examination of individual biological clones of viruses that remain viable amidst a mutagenic treatment evidenced 200-fold reduction in infectivity, with 8-fold increase in mutation frequency [43]. Therefore, the high RHL value in mutagenized populations is likely to reflect a fundamental property of mutant spectra subjected to continuous mutagenesis in which many low fitness genomes are generated. Such genomes, because of the continuous input of new mutations, do not have the opportunity of fitness recovery thus replenishing a low fitness sub-swarm captured by the RHL value. An increase in the proportion of minority (low frequency) mutations has been observed in lethal mutagenesis experiments both in cell culture [19, 20] and *in vivo* [8]. In mutant spectrum expansions that occur under basal mutation rate, RHL is expected to be less abundant because no enhanced mutagenesis jeopardizes opportunities for fitness gain, a tendency documented for RNA viruses when allowed unrestricted replication in a constant environment [31, 32, 44, 45].

Regarding diversity index adequacy to characterize lethal mutagenesis, RHL is followed by the highly correlated incidence-based indices RHL, Hpl, nMuts, PolySites and FAD, the latter probably because its entity level quality prevails under the conditions of our study. In contrast, Mf and Pi, despite being widely used in the description of mutant spectra, exhibit poor correlation with mutagenesis treatment. We also examined by logistic regression the capacity of each index to discriminate between a history of mutagenesis and non-mutagenesis accompanying a mutant spectrum expansion. Sorting of indices by LOOCV error rate placed RHL on top, followed by HpI, PolySites, nMuts, FAD and Shannon. No discriminating capacity is observed for Mf, Pi, Mf.e and Pi.e, in agreement with the poor results of these indices in the association tests. The performed logistic regression has aimed at a more complex scenario, recognizing a mutagenic state independently of population history, where the signal could be blurred and affected by different phases of quasispecies dynamics, either of expansion or contraction of its mutant spectrum.

It could be anticipated that multivariate models such as logistic PCLR and PLSLR might describe the mutagenic effects more accurately than individual indices by adding the contributed predictive capacity of different indices despite its high correlation, in the sense that they could have a higher incidence with samples under mutagenic effect. But no logistic multivariate model beats RHL as a single predictor.

The information we seek to be captured with RHL lies below technical noise. Our approach has consisted in supposing that technical noise affects equally all samples in the experiment and that the distinctive effect would be caused by mutagenesis; that level will include both authentic rare haplotypes and those that are introduced by technical noise.

Deep sequencing has become an important tool to analyze viral populations subjected to mutagenic treatments [8, 22, 46]. The ranking of diversity indices to best characterize mutant spectra subjected to lethal mutagenesis is relevant to a growing body of fundamental and applied studies in virology and microbial genetics in general. Multiple high and low fidelity RNA virus mutants have been characterized [47], and how such mutants modify diversity indices is an open question that may shed light on the biological consequences of altered polymerase fidelity. Also, a large RHL questions the meaning of lethality of mutations in viral and microbial populations [41, 48–51]. Specifically, it is not clear whether the genomes that contribute to the RHL are slow replicators that can participate in evolutionary events, or are dead-end products transiently kept in viral populations by complementation [41, 52]. Ranking of diversity indices may provide also relevant information on the adaptative dynamics under enhanced mutagenesis [53], or the action of mutagenic agents on plant viruses [54]. From the perspective of pharmacology, RHL offers the means to distinguish whether base or nucleoside analogues that display antiviral activities affect viral RNA replication by direct inhibition of polymerase function or by enhanced mutagenesis, thus contributing to clarify uncertainties of drug action. The mechanism of activity of nucleotide analogues has consequences for the types of drug combinations that used together or sequentially can exert a more suppressive antiviral effect [55–58]. Studies with additional viruses, fidelity mutants, and nucleotide analogues are needed to provide a clearer picture of the relevance of different diversity indices to characterize mutant spectra with alternative evolutionary histories. Cellular heterogeneity in important clinical disorders such as cancer parallels the population dynamics and the collective behavior of RNA viruses. Evaluation of a possible applicability of RHL determination as a marker in the mutagenic spectra generated during different cellular tumogeneric processes could also be considered. The present study, however, points towards RHL as a valuable marker for lethal mutagenesis of virus, and emphasizes that the choice of diversity indices to describe mutant spectra is not trivial.

## Materials and methods

### Cells, viruses, infections, and drugs

Huh-7.5 and Huh-7.5 reporter human hepatoma cell lines were grown in Dulbecco’s modified Eagle’s medium, and controlled as previously described [30, 32, 59, 60]. HCV p0 is the parental viral population obtained by electroporation into Huh-7.5-Lunet cells of a transcript of plasmid Jc1FLAG2(p7-nsGluc2A) (a chimera of J6 and JFH-1, genotype 2a) [33], and amplified in Huh-7.5 cells [30]. HCV p100 and HCV p200 resulted from population HCV p0 passaged 100 and 200 times, respectively, in Huh-7.5 reporter cells, as described [32]. Fitness of HCV p100 and HCV p200 relative to HCV p0 was measured in different growth-competition experiments between virus pairs. In initial determinations at a total MOI of 0.03 TCID_50_/cell, HCV p100 fitness was 2.2±0.4 that of HCV p0 [31]. Subsequent determinations gave 1.28±0.34 at a MOI of 0.03 TCID_50_/cell, and 1.10±0.02 TCID_50_/cell at a MOI of 1 TCID_50_/cell; the corresponding values for HCV p200 relative to HCV p0 were 1.33±0.46 and 1.17±0.02, respectively [32]. Infectious HCV was titrated as previously described [32]: serially diluted samples were applied to Huh-7.5 cells in 96-well plates (6,400 cells/well seeded 16 h earlier), and three days post-infection, cells were washed with PBS, fixed with ice-cold methanol, and stained to detect anti-NS5A monoclonal antibody 9E10 [61]. Titrations were performed in triplicate, and titers expressed as TCID_50_/ml. Favipiravir (T-705) (Atomax Chemicals Co. Ltd) and ribavirin (Sigma) were prepared and used as previously described [19, 34, 35]. Their concentrations were chosen to produce comparable inhibition of HCV p0 progeny production.

### RNA extraction, cDNA amplification and deep sequencing

Total intracellular viral RNA was extracted from infected cells using the Qiagen RNeasy kit (Qiagen, Valencia, CA, USA), according to the manufacturer’s instructions. RT-PCR was carried out using AccuScript (Agilent Technologies), with specific oligonucleotide primers (S1 Table) The amplicons covered the following genomic regions: A1, spanning genomic residues 7626 to 7962; A2, residues 7941 to 8257; and A3, residues 8229 to 8653. Negative controls without template RNA were included in parallel to ascertain the absence of cross-contamination by template nucleic acids. PCR products were purified (QIAquick Gel Extraction kit), quantified (Pico Green assay), and analyzed for quality (Bioanalyzer) prior to Illumina MiSeq sequencing.

### Experimental design

The experiment is described schematically in Fig 1A and 1B, as described above. Four factors have been considered. (i) Amplicon, with three levels A1, A2 and A3. Different regions in the ORF are submitted to different functional restrictions. (ii) Base-line passage, with analyses at passes 0, 100 and 200, where starts quasispecies evolution in absence or presence of treatment. (iii) Treatment, with three levels: no drug, favipiravir and ribavirin. (iv) Treatment passages, with analyses at passes 3 and 10. (Fig 1).

### Bioinformatics and statistics

All computations were done in the R environment and language (Team R 2017).

### Fastq data treatment

The fastq files obtained from the MiSeq were subjected to the following treatment. A haplotype-centric data analysis pipeline was developed on targeted samples by amplicons following described procedures [62, 63] adapted to the Illumina MiSeq platform in a paired-end 2x300 mode. It involved the following steps:

- Quality control of fastq files by inspecting profiles of per-site quality, read length and general instrument parameters of quality.
- In paired-end experiments overlap paired reads by FLASh [64] imposing a minimum of 20bp overlapped with a maximum of 10% mismatches.
- Quality profiles of FLASh reads.
- Demultiplex reads by identifying oligonucleotide sequences within windows of expected positions in the sequenced reads.
  By MID (10bp oligonucleotide) distinguishing samples from different patients/origins, only one mismatch is allowed.
  By specific primer (20-30bp oligonucleotides) distinguishing different regions in the genome, and the two strands, up to three mismatches are allowed.
  Trim MIDs and primers.
  As a result, obtain a fasta file by each combination of MID, primer and strand in the run, where the reads were collapsed to haplotypes with corresponding observed frequencies.
- Align haplotypes in each fasta file to the wild type reference sequence or the master sequence in the file (most abundant haplotype) and quality filter.
  Discard haplotypes not covering the full amplicon.
  Discard haplotypes with more than two indeterminations, three gaps or more than 30% differences with respect to the reference.
  Repair accepted indeterminations and gaps as per the reference sequence.
- Intersect haplotypes in both strands with a minimum abundance of 0.1%
  Select haplotypes in both strands with abundances not below 0.1%
  Discard haplotypes unique to one strand.
  Take coverage of haplotypes passing the filter as the sum of reads in both strands.
- The final haplotypes are called consensus haplotypes, and are the basis of the downstream analysis, except for the rare haplotypes load. Final yield 15–25% with respect to raw reads.

The pipeline consists in a set of R [65] scripts using objects and functions in packages Biostrings [66], ShortRead [67], and ape [68].

### Rare haplotypes load (RHL)

It is computed as the fraction of reads in the sample belonging to haplotypes common to the forward and reverse strands with abundance below a given threshold. In the present work 1%, 0.1% and 0.01% have been studied as thresholds, finally taking 1% as the most informative and reliable.

### Down sampling and fringe trimming (DSFT)

To compensate for possible biases in diversity indices due to differences in sample size [27, 29] we used down-sampling followed by fringe trimming, which consists in the following steps: (i) start with fasta files collecting the set of consensus haplotypes with abundance not below 0.1%; (ii) compute the total number of reads in each fasta file in the analysis, and take the minimum as the reference size; (iii) re-size the read number of each haplotype in each fasta file to the reference size; (iv) filter out all haplotypes below 0.2% with 95% confidence. These are the haplotypes and frequencies used in the computation of all diversity indices, except for RHL.

### Association tests

Association tests of all indices with mutagenicity were computed by the non-parametric Wilcoxon signed rank test for paired samples, comparing the diversity values of the mutagenic treatment samples versus the paired control samples, and correcting the p-values for multi-test by the Bonferroni method; we did not distinguish among amplicons. Function wilcox.test with alternate as greater and in paired mode, and p.adjust in package ‘stats’ were used in the computations. Rather than declaring association at any p-value threshold, the models were ranked according to descending order of the standardized effect. The most associated index is considered to be that with the highest effect and still with a low p-value.

### Logistic regression

Logistic regression was used to fit a predictive model of mutagenic activity regardless of the other factors in the design. Computations were performed by function glm in package ‘stats’. To assess the predictive error rate of a fitted model, a sample was considered as under mutagenic effects if the fitted probability was above 0.5. The parameters used in the assessment were: residual deviance, Aikake Information Content (AIC), sensitivity, specificity, area under the ROC curve, and classification error rate. To minimize bias due to overfitting, the cross-validation error rate under 11-fold cross-validation and under leave-one-out cross validation (LOOCV) was used. The models were sorted by increasing order of LOOCV. Function cv.glm in package ‘boot’ was used for the computation of CV error rates [69].

### In silico study

An in-silico analysis was performed to assess the robustness of RHL when compared with Hpl. To this aim, a fasta file of a sample with all haplotypes common to the forward and reverse strands with no previous abundance filter was used to sample and compute its RHL. Then the following steps were repeated 2000 times: (i) take a sample of 40,000 reads from the population; (ii) compute the RHL of this sample, (iii) filter out all haplotypes with abundance below 0.1% and not common to both strands; (iv) DSFT to 20,000 reads with haplotypes not below 0.2% with 95% confidence; (v) count Hpl. In each cycle the number of final reads, haplotypes, and RHL was computed and registered. With the set of 2000 values computed for each fasta file, the mean, median, coefficient of variation (CV), and interquartile dispersion coefficient (QD) were determined. A robust index is that showing the lowest CV and QD.

#### Free available R package

An R package collecting all important functions, the package manual, and tutorial vignettes are freely available from GitHub (https://github.com/VHIRHepatiques/QSutils).

## Supporting information

S1 Table Oligonucleotides used to amplify and sequence HCV p0, HCV p100 and HCV p200 virus subjected to serial passages in the absence or presence of 400 µM favipiravir and 100 µM ribavirin. (DOCX)

S1 Fig Logistic regression plots of mutagenicity over each diversity index considered. A plot for each diversity index. As in Fig 3, bars at the top and bottom depict the values of each diversity index for HCV samples subject to mutagenesis or control. Dots on the logistic curve represent the predicted probability of mutagenesis.
(PDF)

S2 Fig AUC and LOOCV error rates. (top) Barplot with AUC values for each diversity index considered. (bottom) Barplot with LOOCV error rate values for the logistic regression to each single diversity index. All samples included.
(PDF)

S3 Fig AUC and LOOCV error rates. (top) Barplot with AUC values for each diversity index considered. (bottom) Barplot with LOOCV error rate values for the logistic regression to each single diversity index. Samples with a control/treatment of three passes only.
(PDF)

S4 Fig AUC and LOOCV error rates. (top) Barplot with AUC values for each diversity index considered. (bottom) Barplot with LOOCV error rate values for the logistic regression to each single diversity index. Samples with a control/treatment of ten passes only.
(PDF)
