# Supplementary material for: Rare haplotype load as marker for lethal mutagenesis
Source: PLoS One. 2018 Oct 3;13(10):e0204877. doi: 10.1371/journal.pone.0204877 (PMC6169937; doi:10.1371/journal.pone.0204877)
Supplement: S1 Table — (DOCX) [file pone.0204877.s001.docx]

**Supplementary Table 1 (S1).** Oligonucleotides used to amplify and sequence HCV p0, HCV p100 and HCV p200 virus subjected to serial passages in the absence or presence of 400 µM favipiravir and 100 µM ribavirin.

| **Primer name** | **Sequence (5’ - 3’)** | **Sense** | **Position^a^** |
| --- | --- | --- | --- |
| NS5B NakedT1F | TGGTCTACTTGCTCCGAGGAGG | + | 7625 - 7646 |
| NS5B NakedT1R | TTGGCCCCGAATCCATACTTG | - | 7983 - 7963 |
| NS5B NakedT2F | CTTGGAGGAGGCGTGCCAGTTG | + | 7918 - 7940 |
| NS5B NakedT2R | GATACTCCACCCGTTGGGC | - | 8276 - 8258 |
| NS5B NakedT3F | AGCTTCCTCAGGCGGTAATGG | + | 8208 - 8228 |
| NS5B NakedT3R | TCAGGTTCCGCTCGTCCTCCTC | - | 8675 - 8654 |

^a^According to JFH-1, GenBank accession number #AB047639.
