# Supplementary figures and images for: Rare haplotype load as marker for lethal mutagenesis
Source: PLoS One. 2018 Oct 3;13(10):e0204877. doi: 10.1371/journal.pone.0204877 (PMC6169937; doi:10.1371/journal.pone.0204877)

## RHL

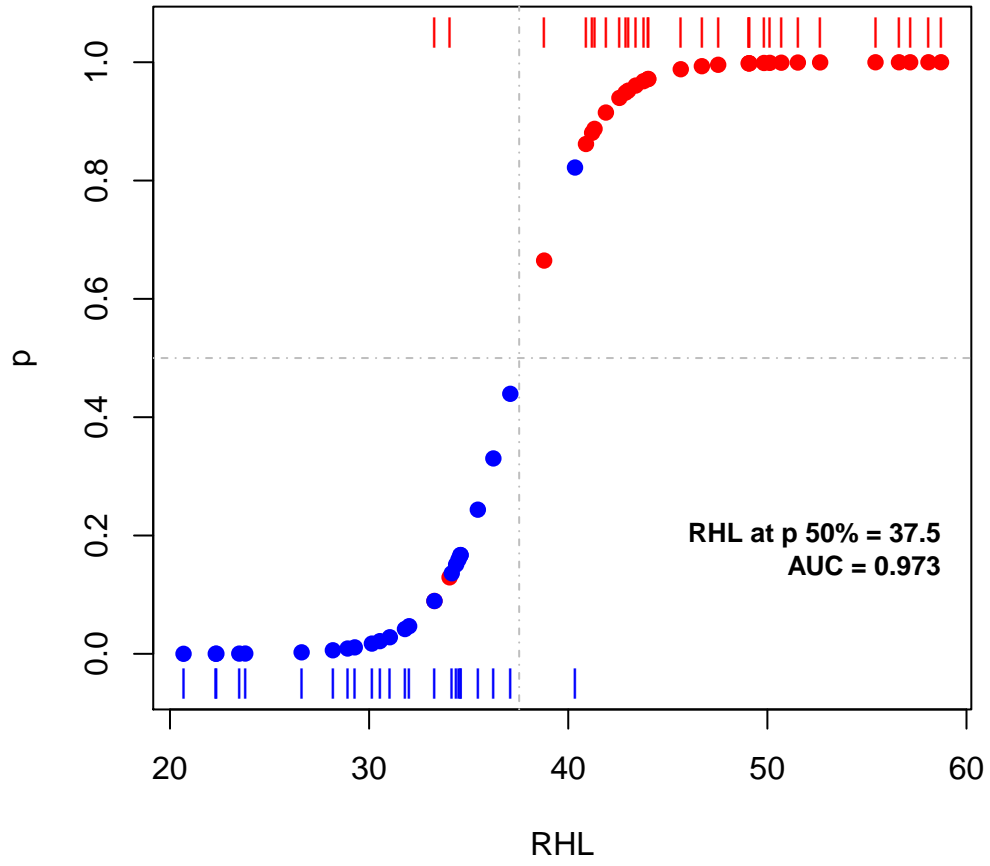

## Hpl

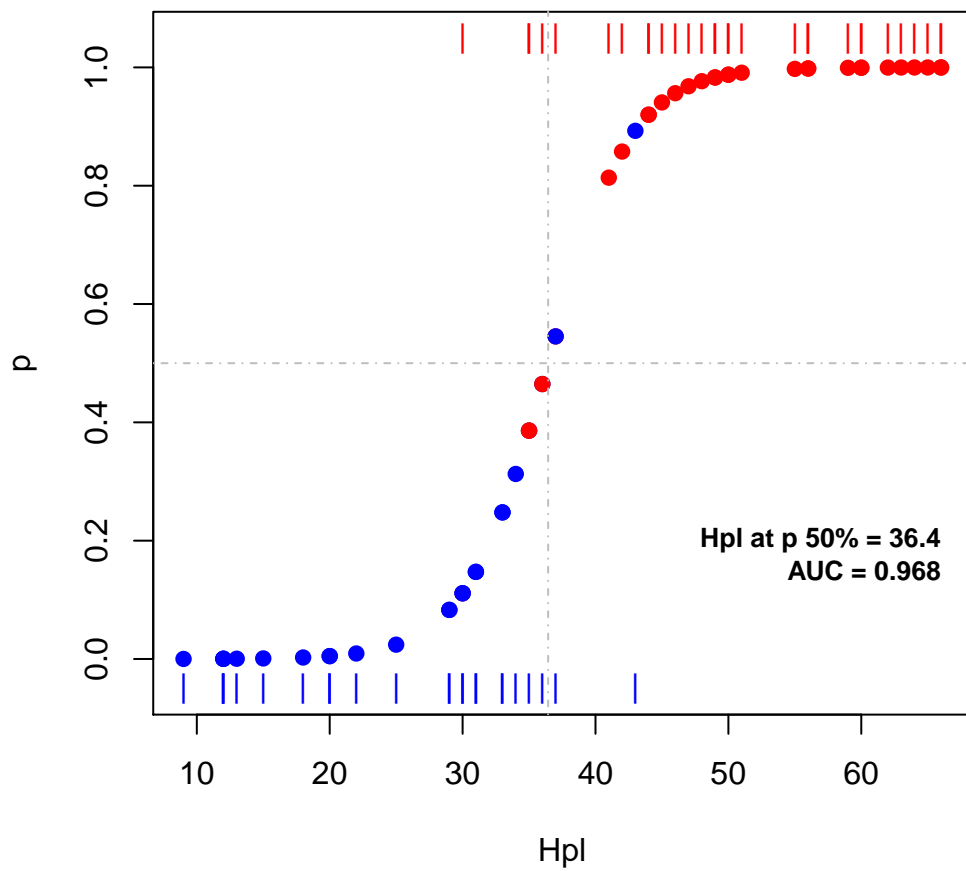

## PolySites

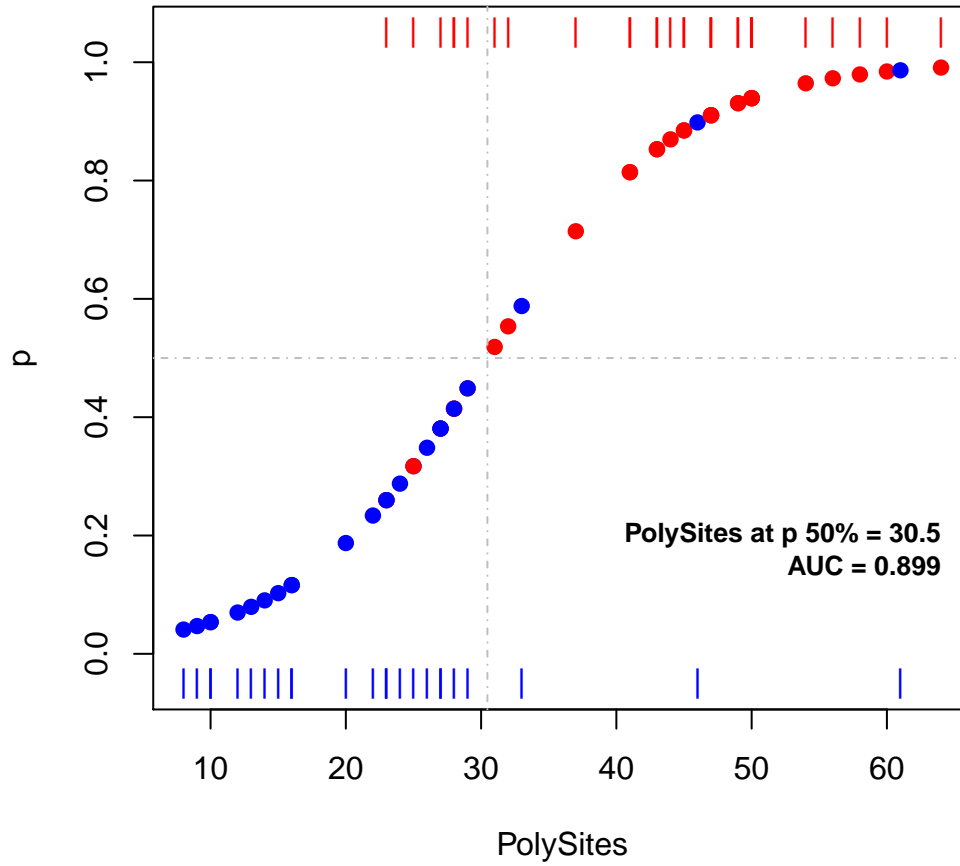

## nMuts

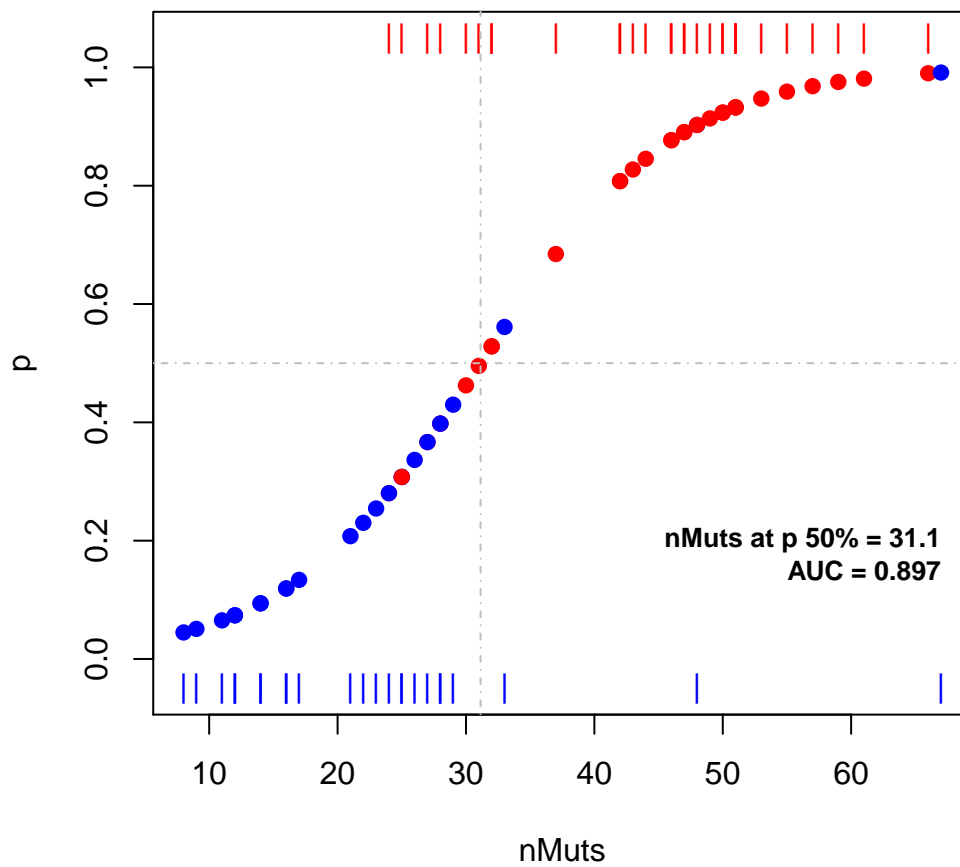

## Shannon

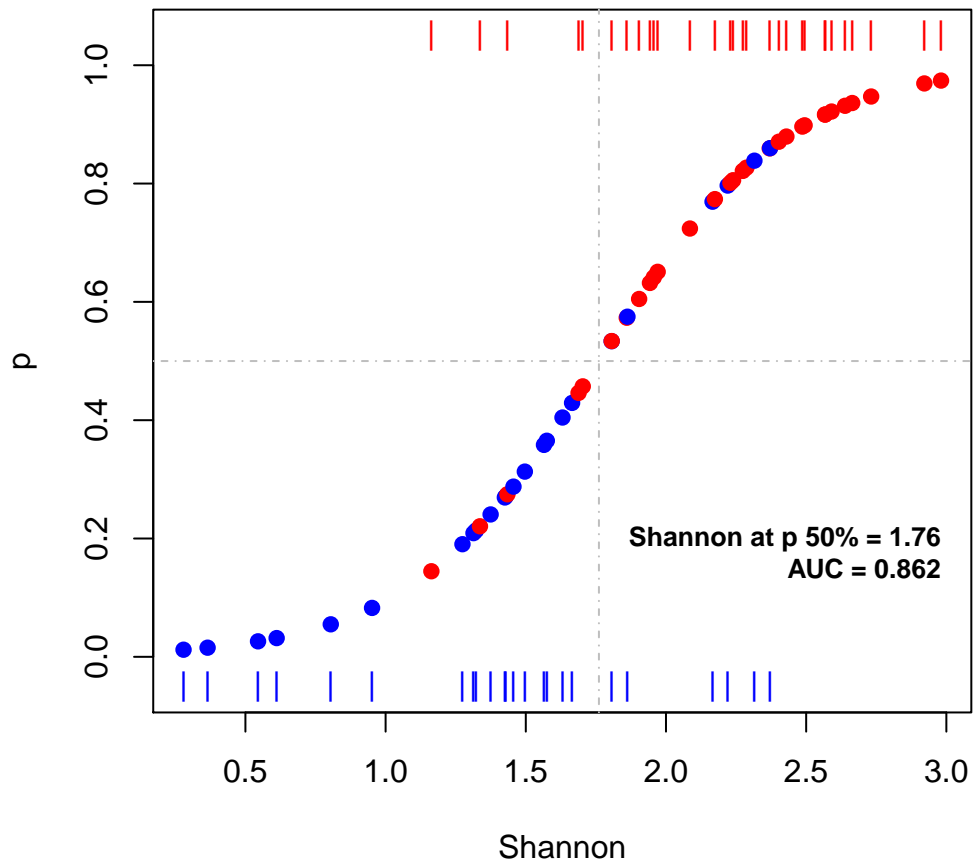

## GiniS

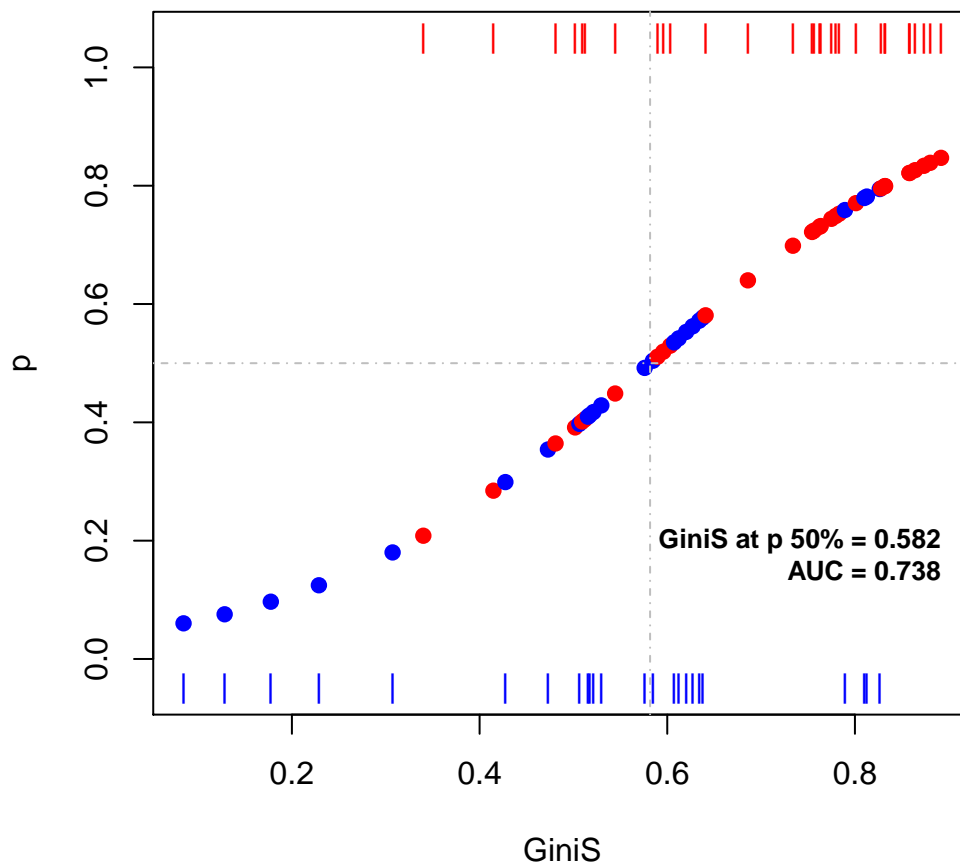

**Mf**

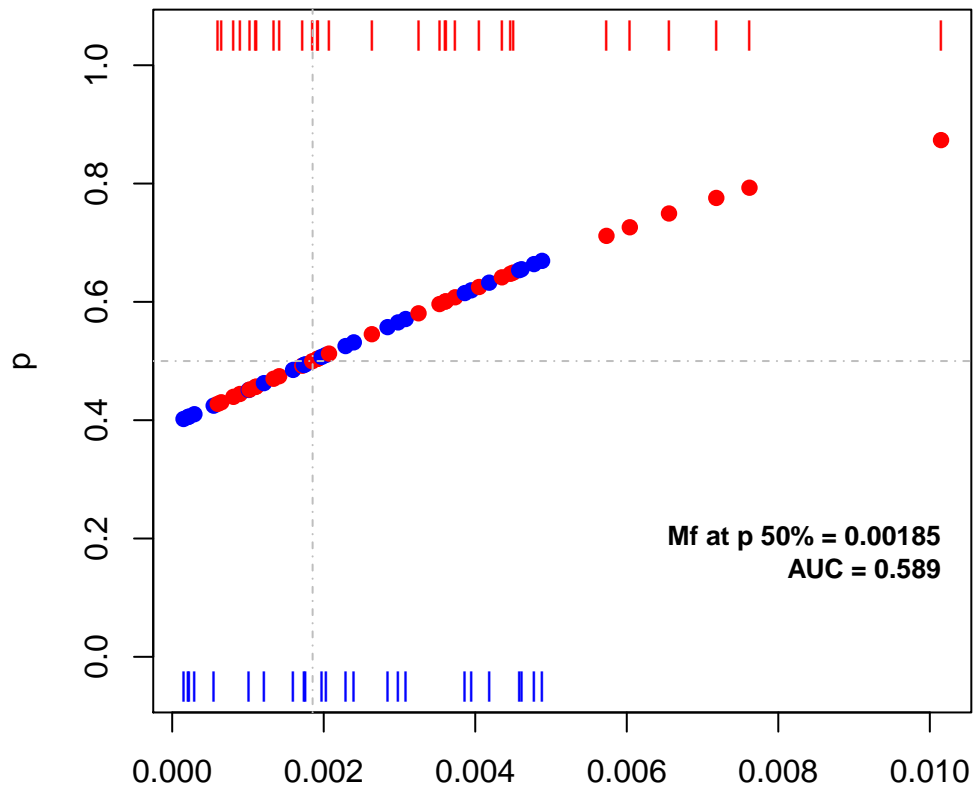

**Mf**

**Pi**

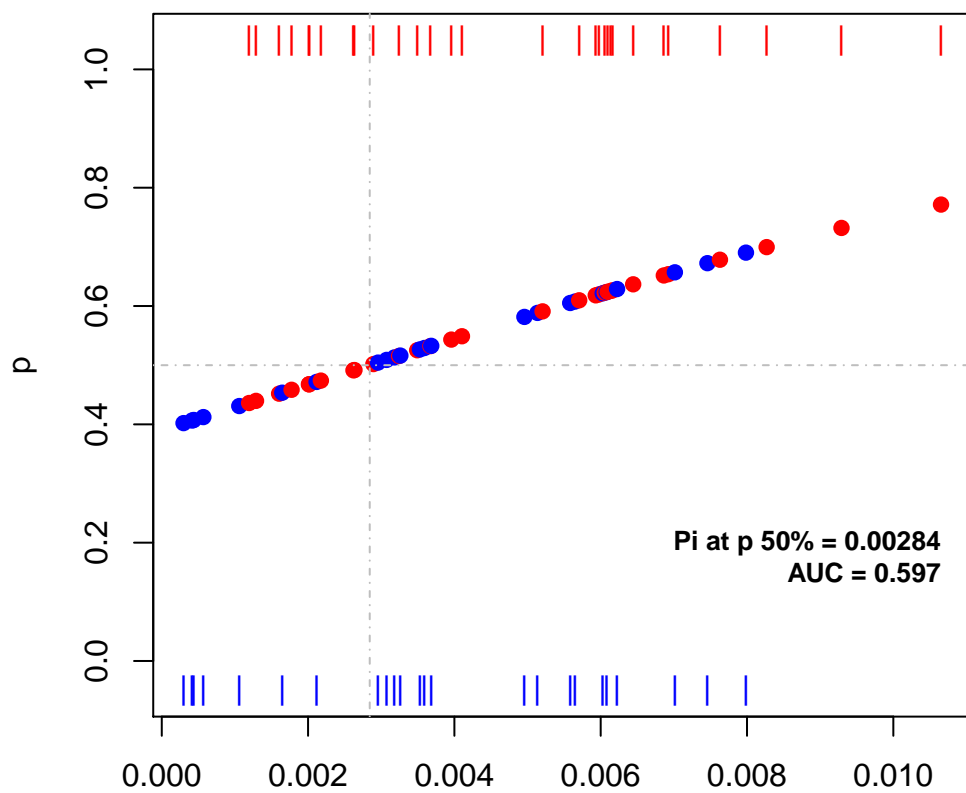

**Pi**

**Mf.e**

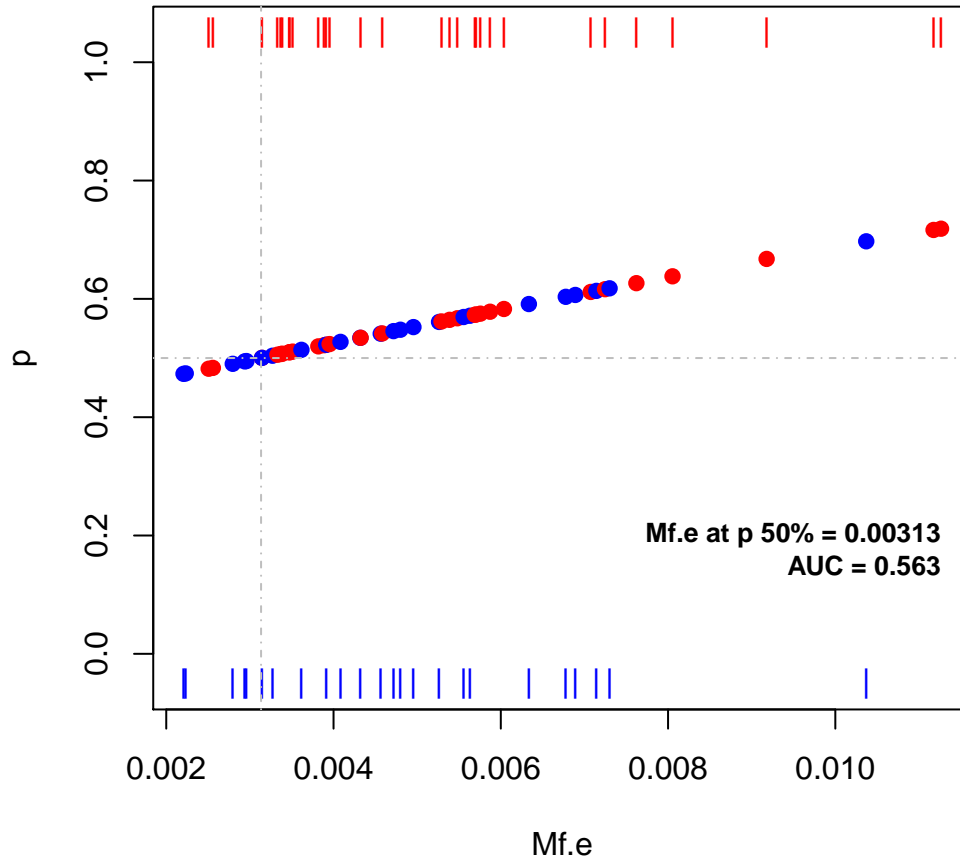

**Pi.e**

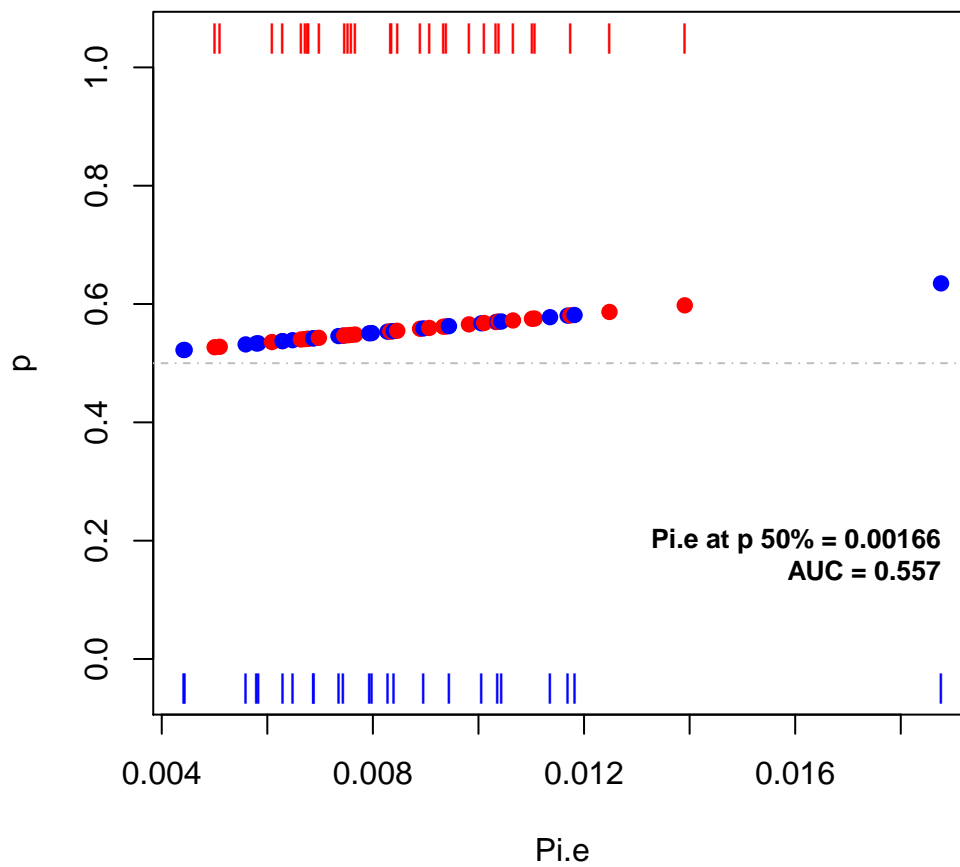

## FAD

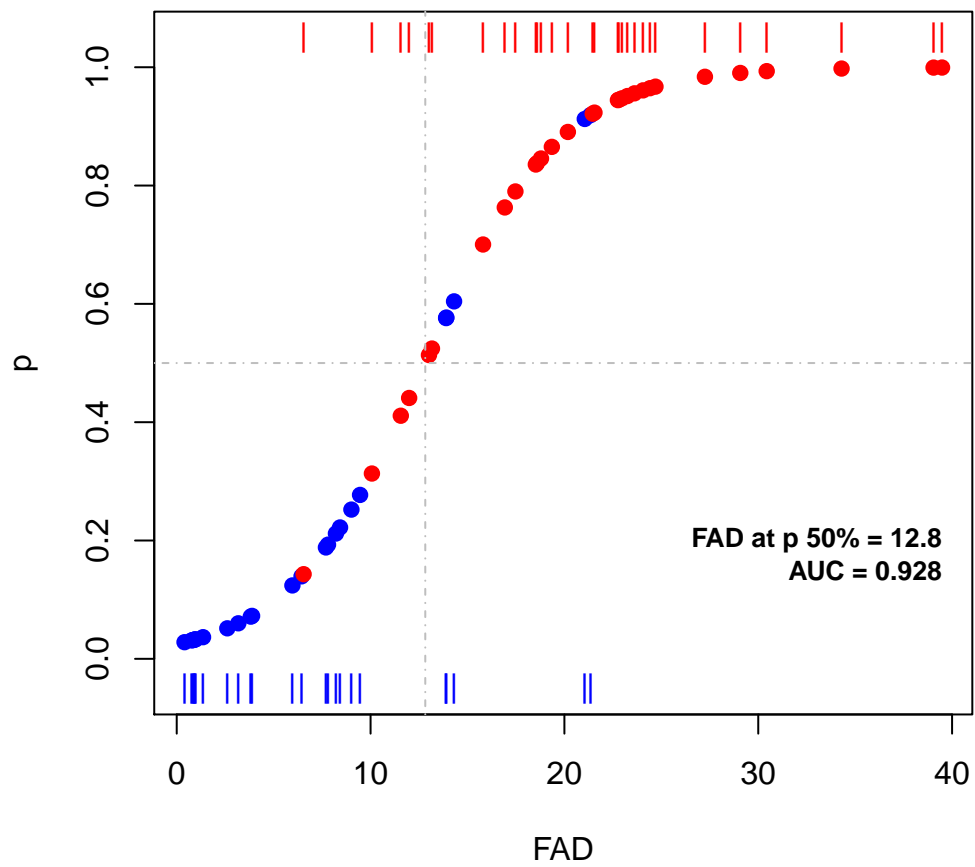

## q1D

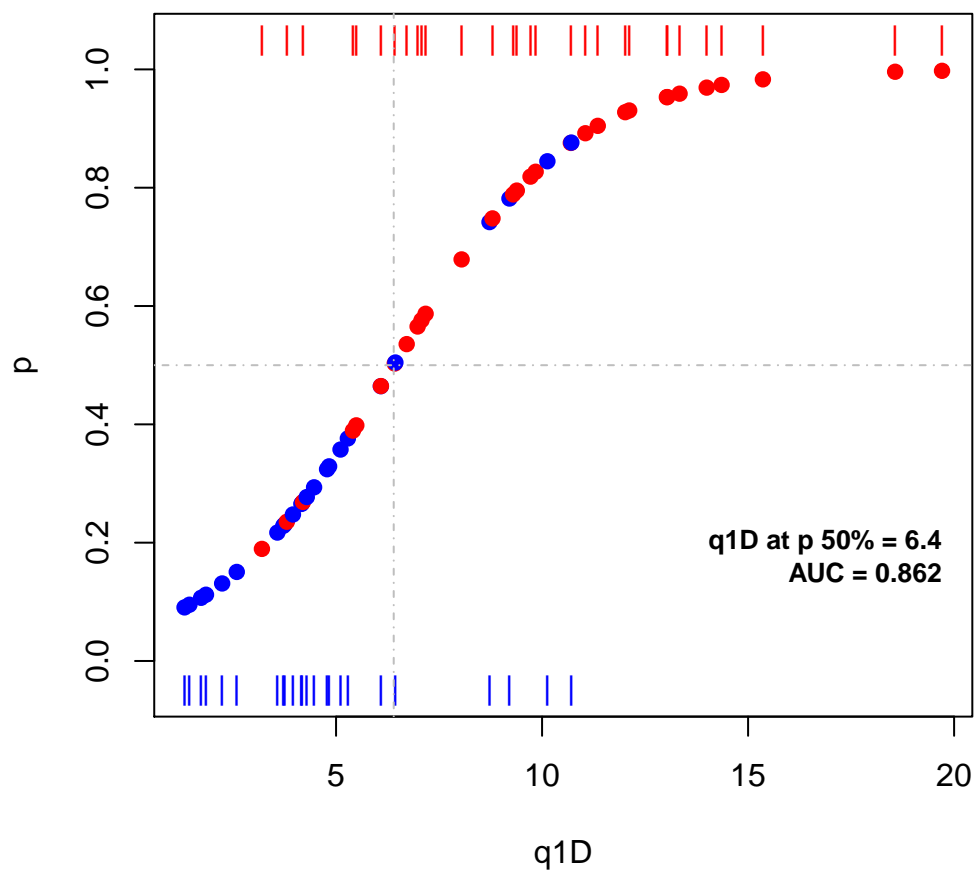

q2D

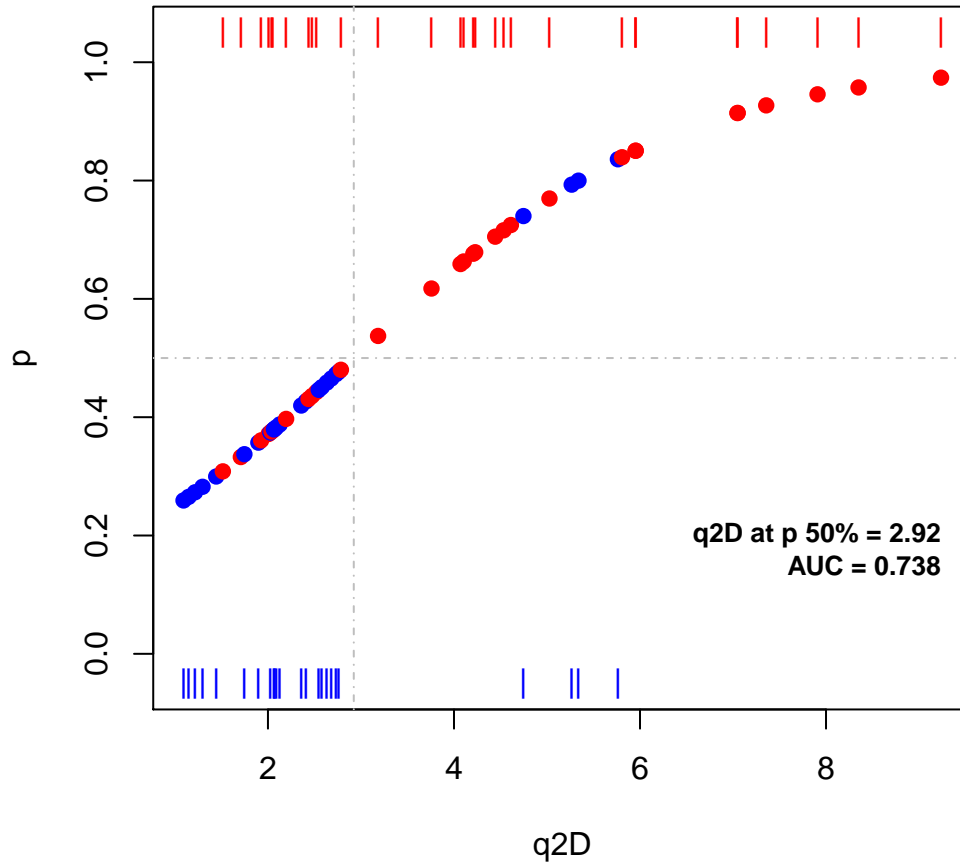

qInfD

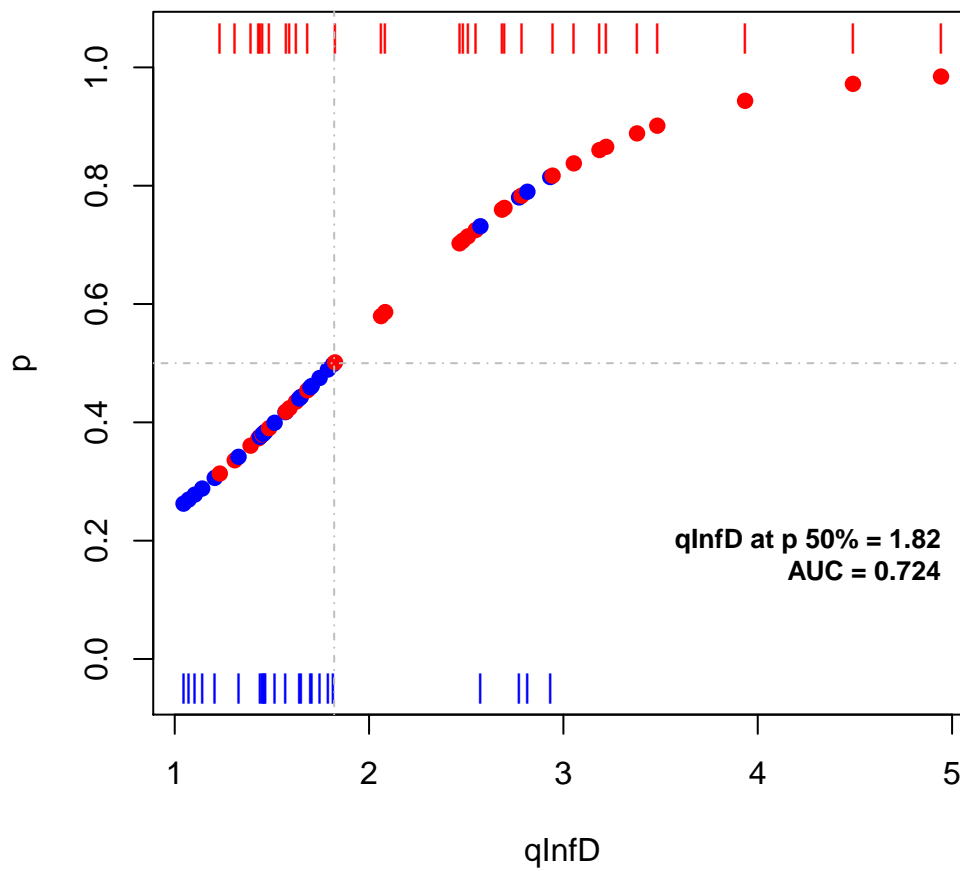

Supplement: S1 Fig — A plot for each diversity index. As in Fig 3, bars at the top and bottom depict the values of each diversity index for HCV samples subject to mutagenesis or control. Dots on the logistic curve represent the predicted probability of mutagenesis. (PDF) [file pone.0204877.s002.pdf]

**Area under ROC curve**

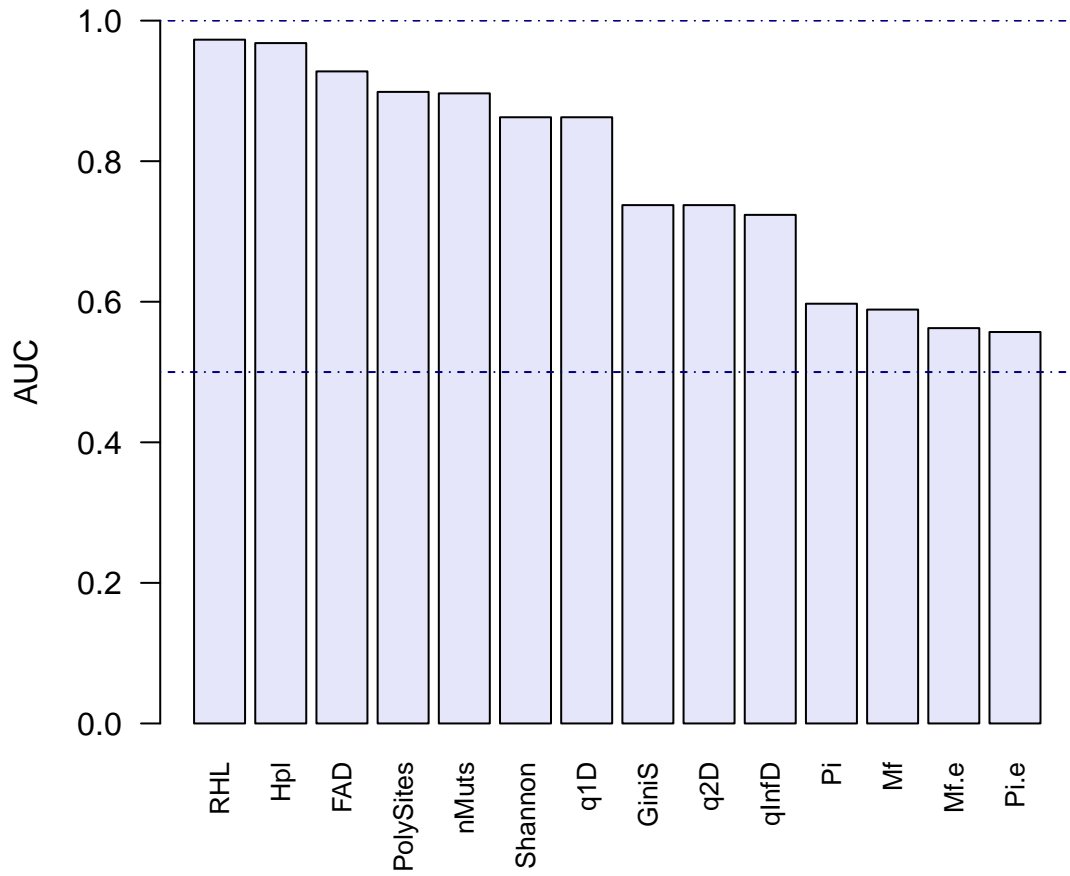

**LOOCV error rate**

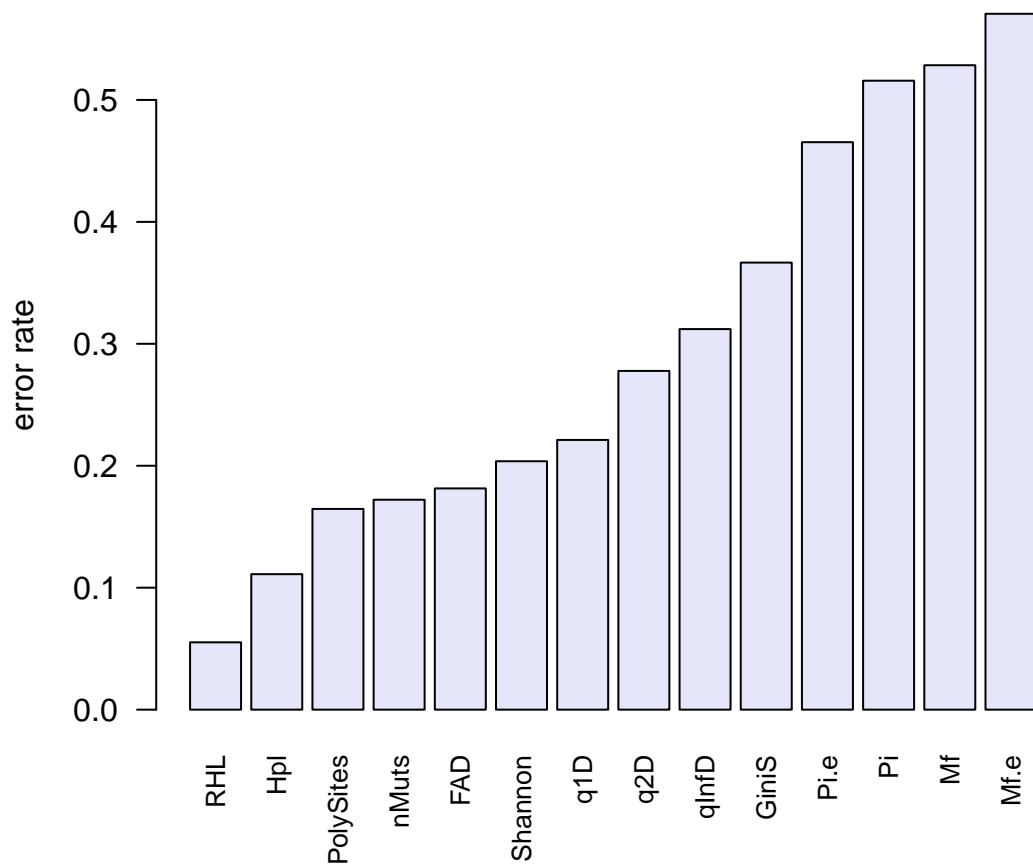

Supplement: S2 Fig — (top) Barplot with AUC values for each diversity index considered. (bottom) Barplot with LOOCV error rate values for the logistic regression to each single diversity index. All samples included. (PDF) [file pone.0204877.s003.pdf]

**Area under ROC curve**

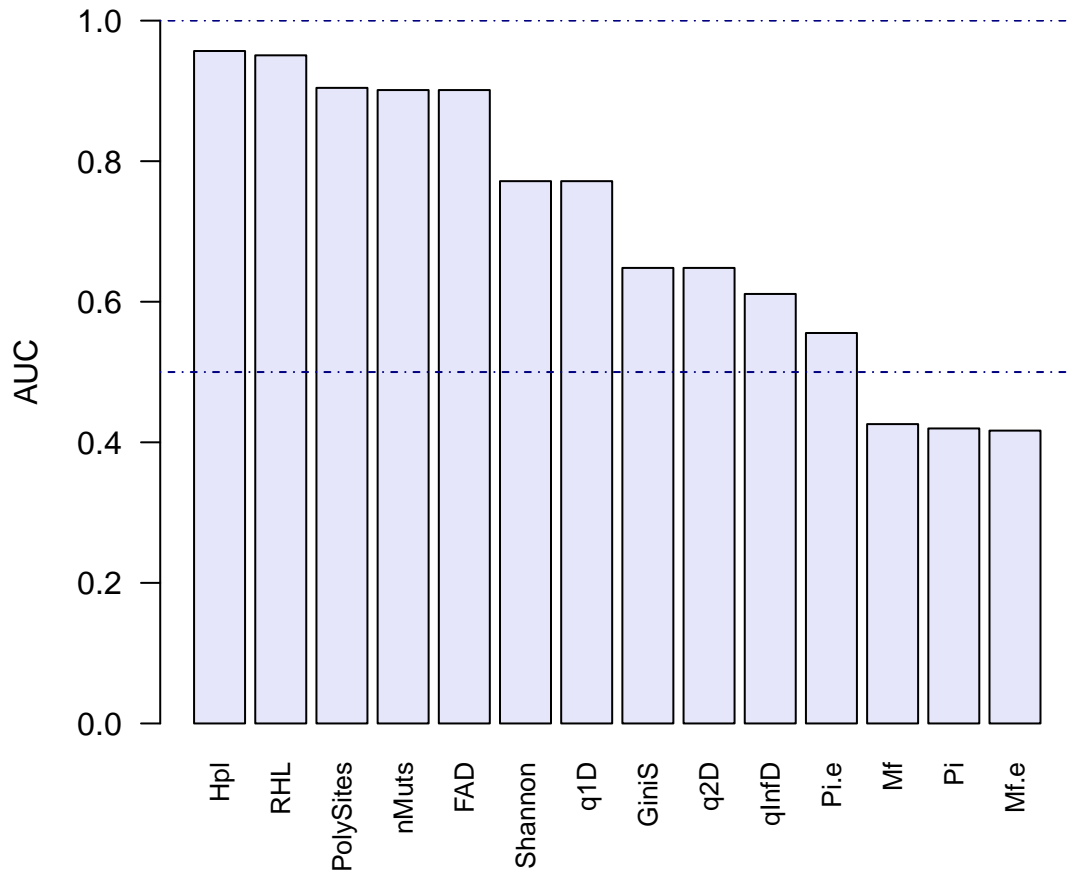

**LOOCV error rate**

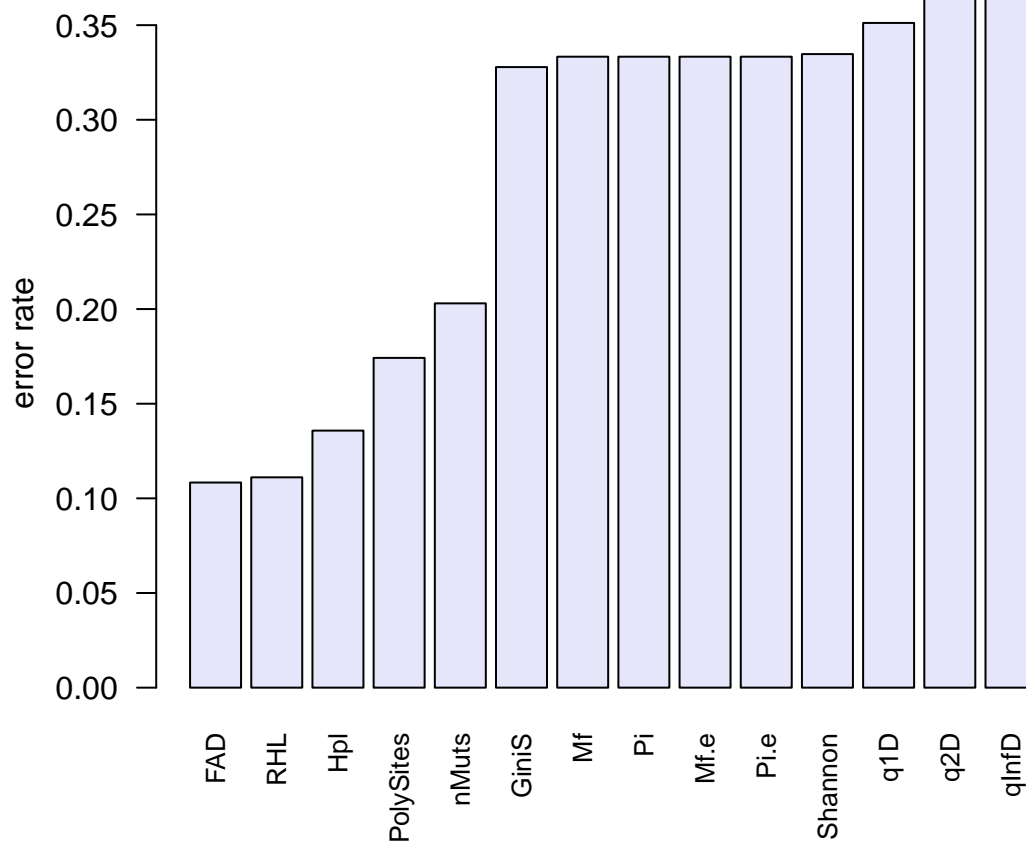

Supplement: S3 Fig — (top) Barplot with AUC values for each diversity index considered. (bottom) Barplot with LOOCV error rate values for the logistic regression to each single diversity index. Samples with a control/treatment of three passes only. (PDF) [file pone.0204877.s004.pdf]

**Area under ROC curve**

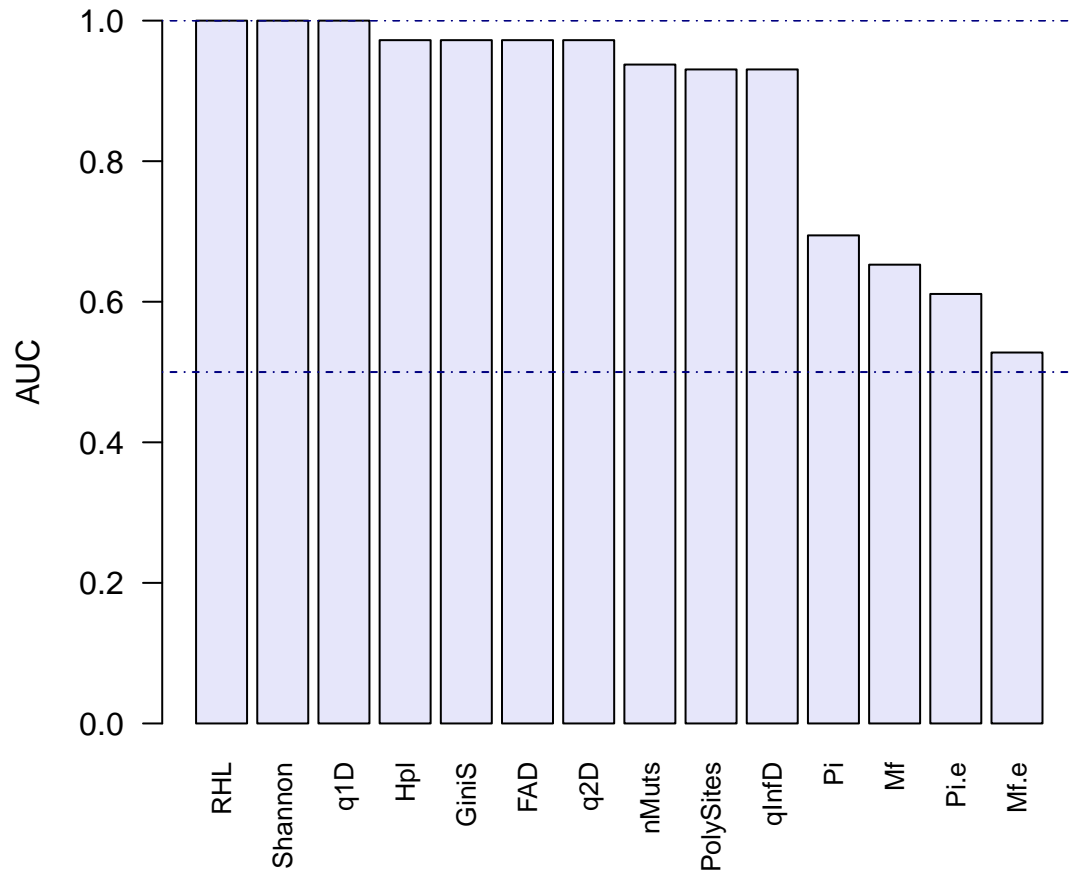

**LOOCV error rate**

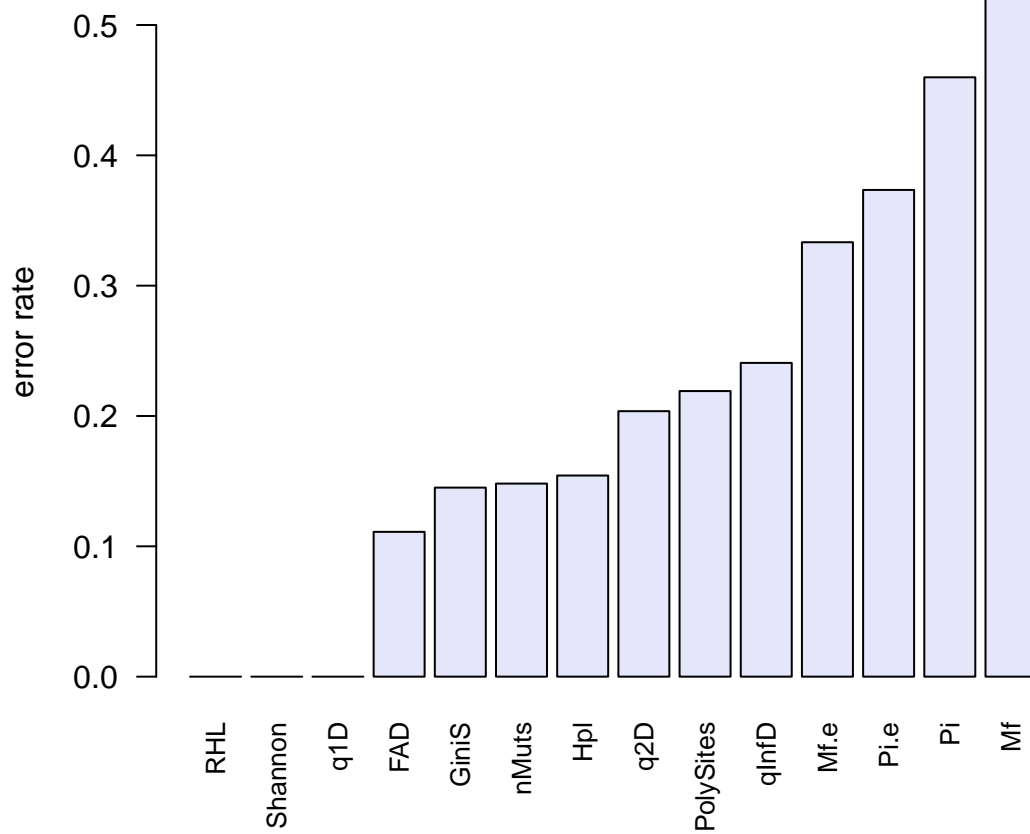

Supplement: S4 Fig — (top) Barplot with AUC values for each diversity index considered. (bottom) Barplot with LOOCV error rate values for the logistic regression to each single diversity index. Samples with a control/treatment of ten passes only. (PDF) [file pone.0204877.s005.pdf]
